# Statement of Retraction: Identification of novel natural inhibitors targeting AKT Serine/Threonine Kinase 1 (AKT1) by computational study

**DOI:** 10.1080/21655979.2026.2728853

**Published:** 2026-10-01

We, the Editors and Publisher of the journal *Bioengineered*, have retracted the following article from publication.

Zhong, S., Hou, Y., Zhang, Z., Guo, Z., Yang, W., Dou, G., Lv, X., Wang, X., Ge, J., Wu, B., Pan, X., Wang, H., Yang, Q., & Mou, Y. (2022). Identification of novel natural inhibitors targeting AKT Serine/Threonine Kinase 1 (AKT1) by computational study. *Bioengineered*, 13(5), 12003–12020. https://doi.org/10.1080/21655979.2021.2011631

Following publication, internal integrity audits revealed concerns around several authorship changes which were made between submission and acceptance of the article. 

We have contacted the authors for an explanation, but we have not received a satisfactory explanation regarding the contributions of authors added or removed at different stages and the rationale for these changes. As determining authorship and contributions is core to the integrity and accountability of published work, we are therefore retracting the article. The authors have been informed of this decision.

We have been informed in our decision-making by our policy on publishing ethics and integrity and COPE guidelines. 

The retracted article will remain online to maintain the scholarly record and will be digitally watermarked on each page as ‘Retracted’. 

